# DNA methylation and differentiation: *HOX* genes in muscle cells

**DOI:** 10.1186/1756-8935-6-25

**Published:** 2013-08-02

**Authors:** Koji Tsumagari, Carl Baribault, Jolyon Terragni, Sruti Chandra, Chloe Renshaw, Zhiyi Sun, Lingyun Song, Gregory E Crawford, Sriharsa Pradhan, Michelle Lacey, Melanie Ehrlich

**Affiliations:** 1 Hayward Human Genetics Program and Tulane Cancer Center, Tulane Health Sciences Center, New Orleans LA, USA; 2 Tulane Cancer Center and Department of Mathematics, Tulane Health Sciences Center and Tulane University, New Orleans, LA, USA; 3 New England Biolabs, Ipswich MA, USA; 4 Institute for Genome Sciences & Policy, Duke University, Durham, NC, USA; 5 Center for Bioinformatics and Genomics, Tulane Health Sciences Center, New Orleans, LA, USA

**Keywords:** Alternative splicing, DNA methylation, Enhancers, H3K4 trimethylation, HOTAIR, *HOX* genes, 5-Hydroxymethylcytosine, Muscle, Myoblasts, Polycomb repression

## Abstract

**Background:**

Tight regulation of homeobox genes is essential for vertebrate development. In a study of genome-wide differential methylation, we recently found that homeobox genes, including those in the *HOX* gene clusters, were highly overrepresented among the genes with hypermethylation in the skeletal muscle lineage. Methylation was analyzed by reduced representation bisulfite sequencing (RRBS) of postnatal myoblasts, myotubes and adult skeletal muscle tissue and 30 types of non-muscle-cell cultures or tissues.

**Results:**

In this study, we found that myogenic hypermethylation was present in specific subregions of all four *HOX* gene clusters and was associated with various chromatin epigenetic features. Although the 3′ half of the *HOXD* cluster was silenced and enriched in polycomb repression-associated H3 lysine 27 trimethylation in most examined cell types, including myoblasts and myotubes, myogenic samples were unusual in also displaying much DNA methylation in this region. In contrast, both *HOXA* and *HOXC* clusters displayed myogenic hypermethylation bordering a central region containing many genes preferentially expressed in myogenic progenitor cells and consisting largely of chromatin with modifications typical of promoters and enhancers in these cells. A particularly interesting example of myogenic hypermethylation was *HOTAIR*, a *HOXC* noncoding RNA gene, which can silence *HOXD* genes in trans via recruitment of polycomb proteins. In myogenic progenitor cells, the preferential expression of *HOTAIR* was associated with hypermethylation immediately downstream of the gene. Other *HOX* gene regions also displayed myogenic DNA hypermethylation despite being moderately expressed in myogenic cells. Analysis of representative myogenic hypermethylated sites for 5-hydroxymethylcytosine revealed little or none of this base, except for an intragenic site in *HOXB5* which was specifically enriched in this base in skeletal muscle tissue, whereas myoblasts had predominantly 5-methylcytosine at the same CpG site.

**Conclusions:**

Our results suggest that myogenic hypermethylation of *HOX* genes helps fine-tune *HOX* sense and antisense gene expression through effects on 5′ promoters, intragenic and intergenic enhancers and internal promoters. Myogenic hypermethylation might also affect the relative abundance of different RNA isoforms, facilitate transcription termination, help stop the spread of activation-associated chromatin domains and stabilize repressive chromatin structures.

## Background

*HOX* genes are a subset of homeobox genes found in four highly conserved gene clusters on different chromosomes. They encode transcription factors essential for determining the vertebrate body axes during embryonic development and for guiding other aspects of prenatal and postnatal differentiation and postnatal homeostasis [1,2]. Probably as a derangement of these normal roles, *HOX* genes are often hypermethylated in cancer [3]. During embryogenesis, the genes within a given *HOX* cluster are activated sequentially in a collinear manner corresponding to the body plan. Because of their pivotal differentiation-linked roles, *HOX* genes must be regulated in a precise spatiotemporal manner, which makes their cell type-specific epigenetics of particular interest. The collinear activation of *HOX* genes during embryogenesis is mediated by the remodeling of chromatin from a repressive to a transcription-permissive state through changes in histone modifications, especially repressive histone H3 trimethylation at lysine 27 (H3K27me3) and activation-associated H3K4 tri-, di- and mono-methylation (H3K4me3, 2 and 1) [4].

We have been studying the epigenetic markers associated with the skeletal muscle lineage, with emphasis on DNA methylation but also incorporating analysis of chromatin epigenetics. DNA methylation is known to vary markedly among different tissues and cell types [5-9]. Human myoblasts (Mb) are an attractive model for analysis of differentiation because they can be efficiently differentiated into very large, multinucleated, postmitotic myotubes (Mt) *in vitro* and can be compared with skeletal muscle tissue, which is largely derived from such myogenic progenitors. The differentiation of Mb to Mt is relevant not only to the formation of skeletal muscle during embryogenesis but also to postnatal repair of muscle [10].

By reduced representation bisulfite sequencing (RRBS) [6], we recently profiled CpG methylation throughout the genome in the muscle lineage using Mb, Mt and skeletal muscle for comparison to 17 nonmyogenic cell cultures and 14 normal nonmuscle tissues [11]. RRBS, which has single-base resolution, detects approximately 5% of genomic CpGs in a wide variety of sequences, namely, gene bodies and intergenic regions; CpG islands, which account for approximately 50% of RRBS-detected CpGs [6], and nonisland sequences; and single-copy and repeated sequences. Using stringent criteria, we identified differentially methylated CpG sites by comparing the set of myoblasts plus myotubes (MbMt) with many diverse nonmuscle cell cultures derived from normal tissues [11]. We similarly mapped CpGs differentially methylated in skeletal muscle vs. nonmuscle tissue. The RRBS-detected CpG sites in Mb and Mt were much more similar to each other than to other cell lineages. When sites with myogenic differential methylation were mapped to the nearest gene and then these genes were examined for related functional terms, homeobox genes were found to be one of the most strongly overrepresented classes among the MbMt-hypermethylated genes.

Homeobox genes include the *HOX* genes, which are oriented in the same direction in a given *HOX* gene cluster so that their intracluster location can be referred to as 5′ or 3′ according to the direction of transcription [12]. This uniform directionality reflects the generation of the archetypal cluster by gene duplication. The ancestral *HOX* gene cluster was in turn replicated to produce four gene clusters. These contain paralogous genes related by sequence similarity and intracluster position and were assigned to the same number group. Paralogous *HOX* genes have many similarities in function but can also display distinct functionality [12,13].

The *HOXA*/*Hoxa* cluster is implicated in regulating mouse limb bud development (especially *Hoxa9*–*Hoxa13*) [14]. *Hoxa9* and *Hoxa10* are expressed in the murine C2C12 Mb cell line and in limb muscles during embryogenesis and postnatally, but *Hoxa10* was repressed during muscle regeneration following injury [15]. Targeted disruption of *Hoxa13* increased the level of expression of the myogenic transcription factor MyoD in embryonic murine forelimb [16]. *Hoxa1* coordinates the expression of other *Hoxa* genes in murine embryonic stem cells upon induction by retinoic acid, leading to demethylation of H3K27me3 [17]. *HOXA/Hoxa* genes are expressed in some postnatal lineages, including hematopoietic cells [18], adult lung [19] and endometrium [20]. Unlike *HOXA/Hoxa* genes, *HOXB/Hoxb* genes are not detectably expressed in murine limb muscle during embryogenesis [15]. However, *Hoxb5* is implicated in determining limb positions along the anteroposterior axis [21]. Among other functions, *HOXB/Hoxb* genes are likely to play a role in lung development [19] and hematopoiesis [22].

Murine *Hoxc* genes are also expressed in the skeletal muscle lineage, including *Hoxc12* in embryonic myoblasts [23] and *Hoxc9–Hoxc13* in the embryonic muscle hindlimb, but not in the forelimb [15]. *Hoxc6*, *Hoxc9*, *Hoxc10* and *Hoxc11* are expressed in murine C2C12 Mb and Mt [15] and during the formation of other organ systems, such as the nervous system [24]. Among the postnatal tissues with specific expression of *HOXC/Hoxc* genes are muscle [15], lymphocytes [25], mammary glands [26], skin and keratinocytes [27]. *HOXD/Hoxd* genes, like *HOXA/Hoxa* genes, appear to play especially important roles in limb and digit formation [14,28] as well as in the development of other organs, such as the formation of the terminal regions of the digestive and urogenital tracts [12]. However, *Hoxd11* is expressed in embryonic muscle, but not in postnatal muscle or C2C12 Mb or Mt [15].

Differential expression of *HOX/Hox* genes in a spatially and temporally specific manner is associated with chromatin modification [29-31], expression of ncRNAs (including miRNAs) in cis or trans [32-34], long-distance enhancers outside the *HOX* clusters as well as local enhancers [35] and three-dimensional chromatin architecture [4,36]. Studies of specific *HOX/Hox* genes have revealed tissue-specific DNA methylation, which is likely to help lock in complicated expression patterns for *HOX* genes and possibly help to establish these expression patterns [37-40]. In a whole-genome analysis of DNA methylation, the four *HOX* gene clusters were found to be hypomethylated in human embryonic stem cells (ESCs) relative to fibroblast-like derivatives of ESC, neonatal foreskin fibroblasts and blood monocytes [41]. To the best of our knowledge, the present study is the first to use single-base resolution profiling of DNA methylation to investigate all the *HOX* clusters in a wide variety of normal cell cultures and tissues. We also correlated DNA epigenetic differences with differential chromatin epigenetics and gene expression. We found that the variety of *HOX* genes’ functions is reflected in their developmentally associated DNA methylation patterns, which had diverse relationships with gene expression.

In addition, we examined whether DNA hypermethylation in myogenic progenitor cells involves 5-methylcytosine (5mC) or 5-hydroxymethylcytosine (5hmC) because they cannot be distinguished by RRBS or most other types of DNA methylation analysis [42]. In mammalian DNA, 5hmC is the sixth genetically programmed base. It is usually very much less abundant than 5mC and serves as an intermediate in DNA demethylation as well as a stable DNA base [43,44]. Increases in 5hmC and decreases in 5mC have been reported in *HOXA1* and *HOXA2* upon induction of differentiation of the NT2 embryonal carcinoma cell line by retinoic acid, which derepresses *HOX* genes in a collinear manner [45]. Discriminating between 5mC and 5hmC is important because they seem to typically play very different roles in the control of gene expression, usually repression at cis-acting transcription control elements for 5mC and activation at enhancers for 5hmC [42,46]. Therefore, we quantified 5mC and 5hmC at five representative CpG sites in the four *HOX* clusters of muscle and nonmuscle samples by enzymatic assay.

## Results and discussion

### Myogenic DNA hypermethylation at *HOXD* genes vs. H3K27me3 in many cell types

To identify myogenic differential methylation in *HOX* gene clusters, we analyzed RRBS data from the ENCODE project ([11]; http://genome.ucsc.edu/; DNA methylation by RRBS; HudsonAlpha Institute for Biotechnology, Huntsville, AL, USA). The methylome profiles that we used were generated from our Mb and Mt samples plus 16 other types of cell culture and skeletal muscle plus 14 types of normal tissue. The Mb samples were derived from biopsies, and aliquots were differentiated to Mt. Importantly, all had been characterized immunocytochemically as previously described [11]. The nonmuscle cultures were untransformed cells, with the exception of lymphoblastoid cell lines (LCLs). We determined significant myogenic hypermethylation or hypomethylation using stringent criteria, namely, at least a 50% difference in methylation in Mb and Mt (as a set, MbMt) vs. the nonmyogenic cell cultures or in skeletal muscle tissue vs. nonmuscle tissue at a significance level of *P* < 0.01 using fitted binomial regression models at each monitored CpG site [11]. This analysis involved our recently developed algorithm that adjusts single-site *P* values for coverage score and sample size. We then plotted the sites with myogenic differential methylation to the nearest gene and subgene region as illustrated for *HOX* genes in Additional file 1. All our references to myogenic differential methylation met the above requirements for statistical significance.

**Figure 1:**
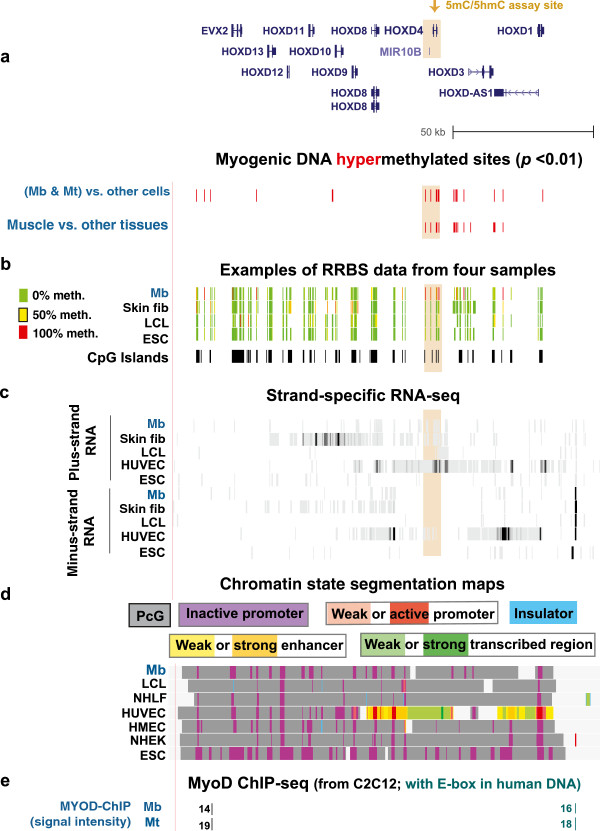
** Myogenesis-associated hypermethylation in the 3' half of the *HOXD *gene cluster, which displayed polycomb silencing in most cell types. (a)** Red bars, the 55 CpG sites significantly hypermethylated in Mb plus Mt vs. 16 types of non-muscle-cell cultures and 61 CpG sites significantly hypermethylated in skeletal muscle tissue vs. 14 types of nonmuscle tissues in the chr2:176,921,692 -177,074,604 region. At this scale, many differentially methylated sites cannot be discriminated. **(b)** Examples of RRBS data **(a)**. Using an 11-color semicontinuous scale (see color guide), these tracks indicate the average DNA methylation levels at each monitored CpG site from the quantitative sequencing data (ENCODE/HudsonAlpha Institute for Biotechnology). Data are shown for only a few of the cell culture samples evaluated for this study. Skin fib, neonatal foreskin fibroblasts. **(c)** Strand-specific RNA-seq profiling at the *HOXD* gene cluster for Mb, neonatal foreskin fibroblasts, HUVEC and ESC. Each track displays the signal from RNA-seq (ENCODE/Cold Spring Harbor Laboratory, Cold Spring Harbor, NY, USA) from these cell cultures. The vertical viewing range for the strand-specific RNA-seq was 1 -100 in the UCSC Genome Browser for this and subsequent figures unless otherwise noted. Tan highlighting, the *HOXD4* region shown in Additional file 2. **(d)** The predicted type of chromatin structure in subregions of the *HOXD* gene cluster is displayed in chromatin state segmentation maps (ENCODE/Broad Institute, Cambridge, MA, USA) based mostly on histone modifications [54]. The predicted local chromatin states are shown with the indicated colors. PcG, polycomb group protein -associated H3K27me3. **(e)** MyoD binding from C2C12 ChIP-seq [59] and identification of orthologous human sequences. The relative binding strength is indicated, and sites shown in blue overlapped CAGCTG, which is present in approximately 75% of Myod ChIP-seq peaks and is part of the degenerate consensus sequence for MyoD binding [59].

In the *HOXD* gene cluster, many sites were hypermethylated in the MbMt set vs. nonmuscle cell cultures or in skeletal muscle tissue vs. nonmuscle tissues, as shown in Figure 1a. Figure 1b displays the coverage of RRBS in this region by exhibiting DNA methylation data tracks from the UCSC Genome Browser for representative samples. One of the subregions with the most myogenic hypermethylation in both progenitor cells and tissues was in the vicinity of *HOXD4* and had 38 MbMt-hypermethylated sites and 33 skeletal muscle-hypermethylated sites (Figure 1a, tan highlighting, and Additional file 2). The two clusters of MbMt-hypermethylated sites in the *HOXD4* upstream region surround a retinoic acid-sensitive mesodermal enhancer [47] and are near the adjacent *MIR10B* gene (Figure 1), whose methylation was implicated in gene silencing in cis in gastric cancer [32]. Both DNA methylation and H3K27me3 were seen at the *MIR10B* promoter region in human mammary epithelial cells (HMEC) in a previous study [48] as well as in the present study (Figure 1 and Additional file 2). Our analysis of RNA-seq data (ENCODE/California Institute of Technology; http://genome.ucsc.edu/; [49]) by Cufflinks [50], a program which evaluates RNA-seq profiles to determine steady-state amounts of different RNA isoforms, indicated that human umbilical vein endothelial cells (HUVEC) expressed this gene abundantly, whereas less than 200 times as much *HOXD4* RNA was detected in Mb, epidermal keratinocytes (NHEK), lung fibroblasts (NHLF), ESC and an LCL (Additional file 1). Only HUVEC did not have the repressive polycomb group chromatin marks at *HOXD4* and throughout most of the *HOXD* cluster (Figure 1d). However, the predominant, 5.1-kb HUVEC transcript began upstream of *HOXD4* near the *MIR10B* gene and extended past the 3′ end of *HOXD4*. A second, noncoding transcript was seen in HUVEC (ENST00000465649), whose transcription begins within the single *HOXD4* intron. The myogenic intragenic hypermethylated sites in *HOXD4* surround or overlap this alternative transcription start site (TSS; pink triangle, Additional file 2). Myogenic hypermethylation of the intron might help suppress the use of a secondary, intronic promoter.

**Figure 2:**
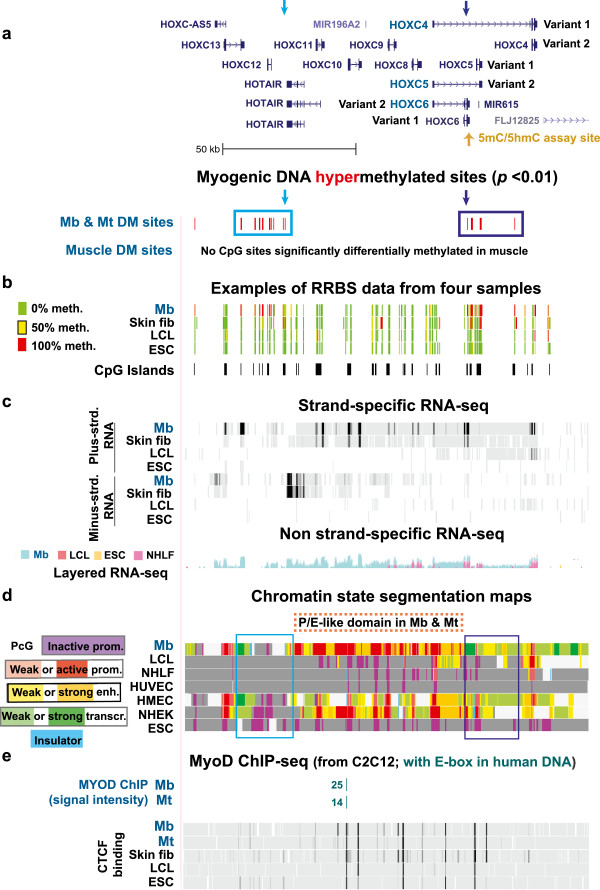
**Myogenic hypermethylated sites at both ends of the *HOXC *gene cluster, which was preferentially transcribed in myogenic cells. (a)** 119 CpG sites with significant hypermethylation in Mb plus Mt vs. 16 types of non-muscle-cell cultures at chr12:54,318,064–54,468,880. **(b)** Representative RRBS tracks with the location of CpG islands beneath them. **(c)** Strand-specific RNA-seq profiling (as in Figure 1) for the *HOXC* gene cluster and standard RNA-seq (not strand-specific; ENCODE/California Institute of Technology). The layered RNA-seq shows the superimposed profiles from Mb, LCL, ESC and NHLF cells in the indicated color code. **(d)** Chromatin state segmentation analysis as in Figure 1. **(e)** MyoD binding site profiles as in Figure 1 and CTCF binding from ChIP-seq profiling of the indicated cell types (ENCODE/Broad Institute). Arrows and empty boxes denote features mentioned in the text.

**Figure 3:**
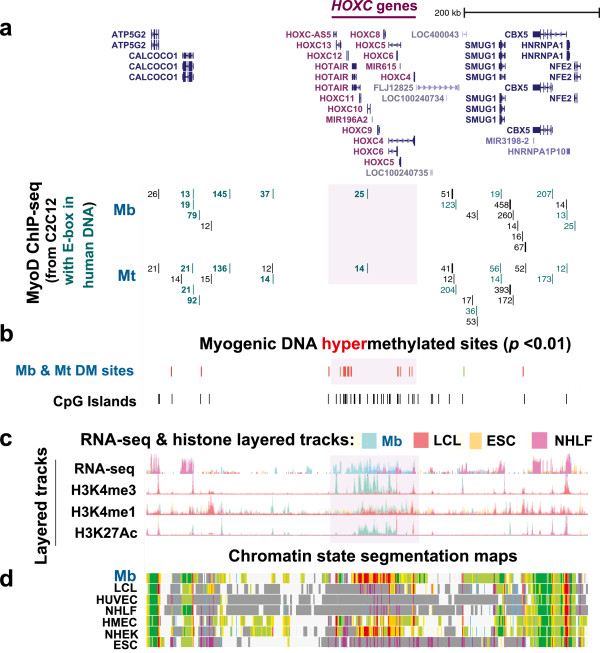
**Myogenic hypermethylation, enrichment in CpG islands and extensive myogenesis-associated transcription localized to the 151-kb *HOXC *cluster. (a)** MyoD binding profiles show that inferred MYOD binding sites form a distant border on both sides of the *HOXC* cluster. MYOD binding sites were extrapolated and are depicted as in Figure 1. The visualized chromosomal region from the UCSC Genome Browser for this figure is chr12:54,052,006–54,706,150 (654 kb). **(b)** 119 MbMt-hypermethylated sites and the distribution of CpG islands. **(c)** Layered RNA-seq track as in Figure 2 with additional layered tracks for H3K4me3, H3K4me1 and H3K27Ac by ChIP-seq (ENCODE/Broad Institute). **(d)** Chromatin state segmentation analysis as in Figure 1. The pink-highlighted region is the *HOXC* gene cluster shown in Figure 2.

**Figure 4:**
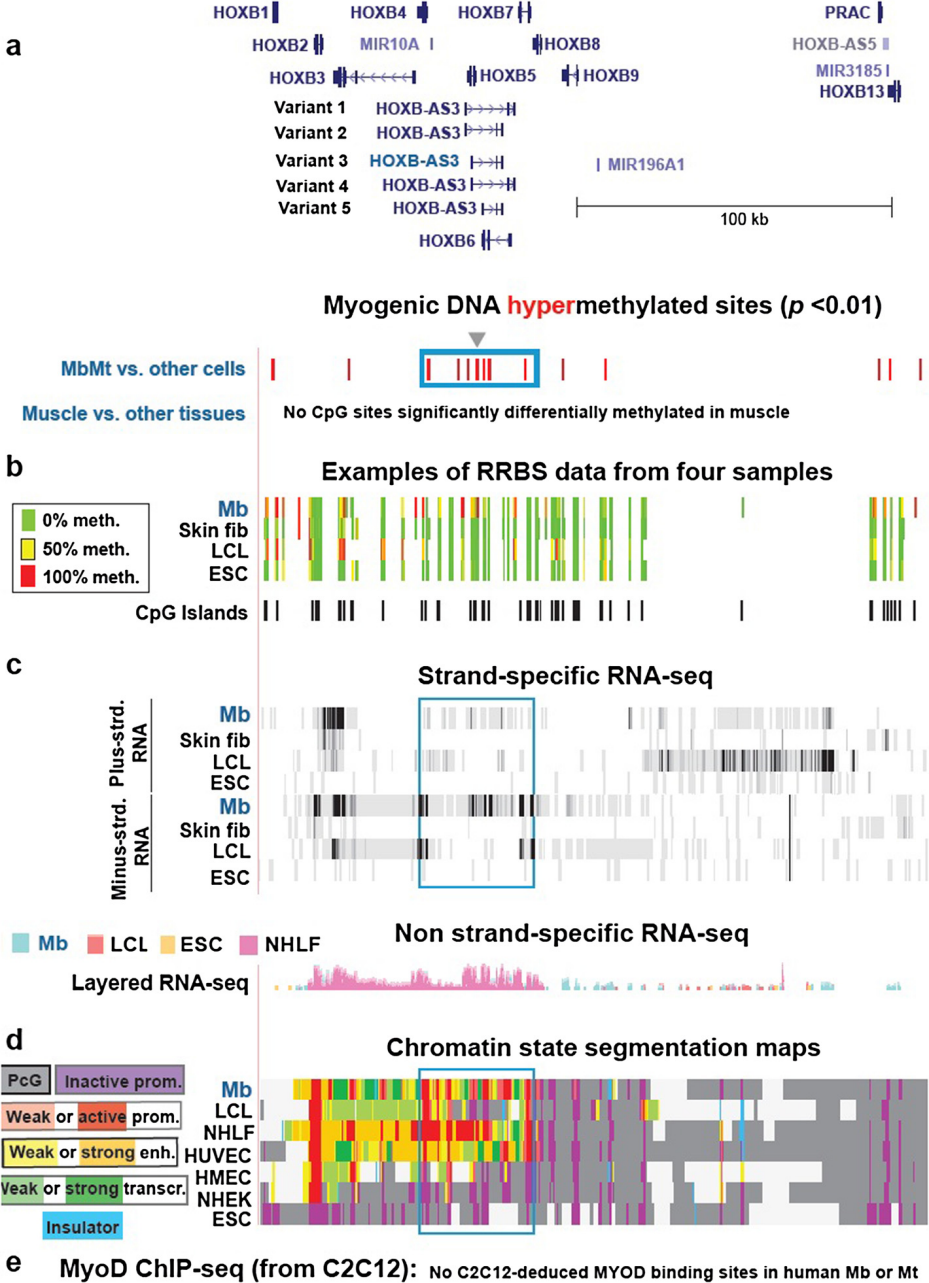
**Myogenic hypermethylation in the central region of the *HOXB *gene cluster, which is preferentially transcribed in myogenic cells. (a)** 88 MbMt-hypermethylated sites in the chr17:46,602,904–46,814,469 region. **(b)** Examples of RRBS data. **(c)** Strand-specific RNA-seq as in Figure 1, except that the vertical viewing ranges were 1–10 for the plus strand was and 1–100 for the minus strand. **(d)** Chromatin state segmentation analysis. **(e)** The MyoD binding site track shows no C2C12-extrapolated MYOD sites in this region. Arrows, empty boxes and the triangle denote features mentioned in the text.

**Figure 5:**
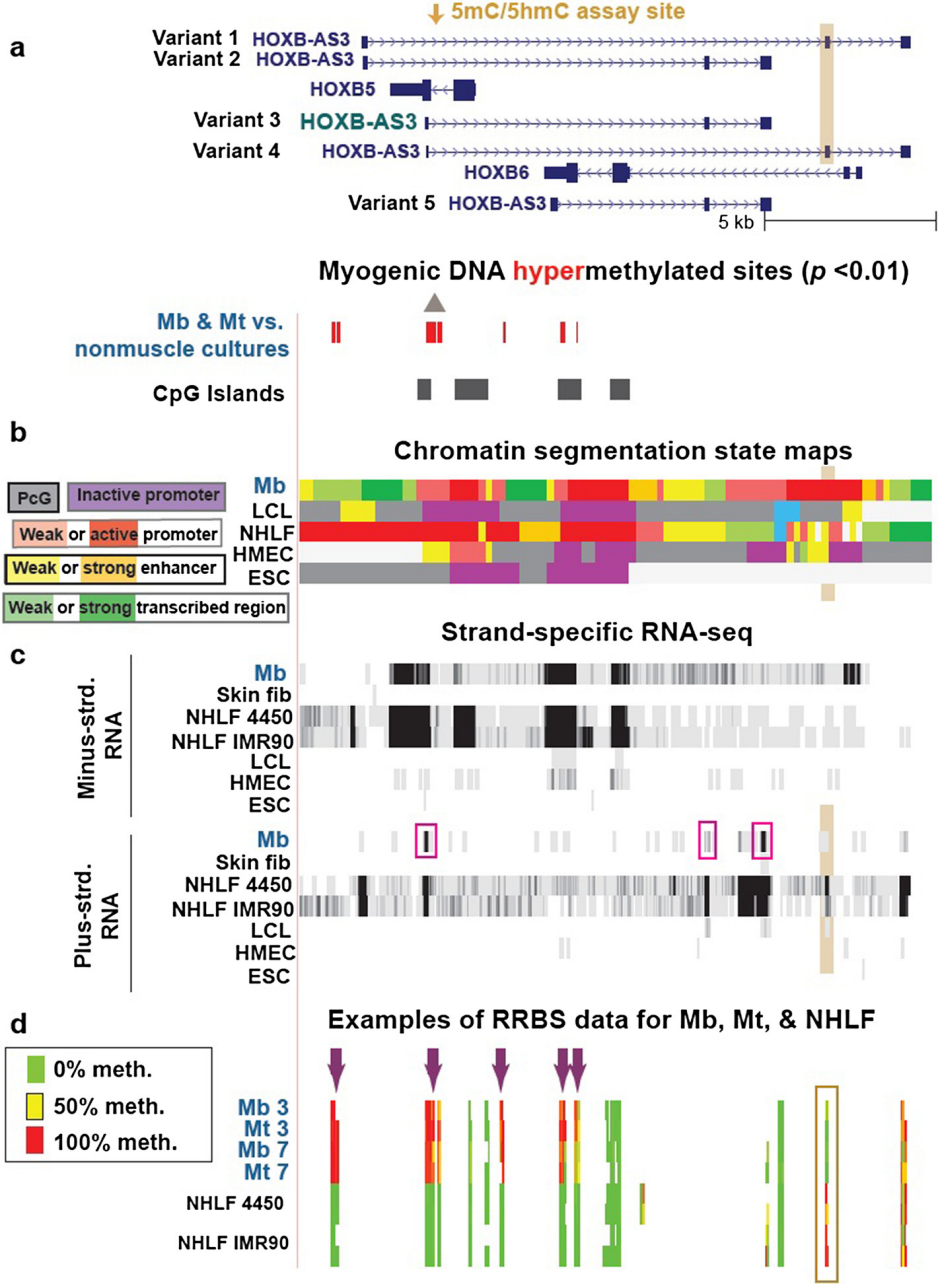
**Cell type–specific differences in DNA methylation and transcription in the region containing *HOXB5*, *HOXB6 *and *HOXB-AS3 *variant genes. (a)** 42 MbMt-hypermethylated sites in a subregion of *HOXB* (chr17:46,665,998–46,684,371). **(b)** Chromatin segmentation state maps. **(c)** Strand-specific RNA-seq as in Figure 4. The pink boxes indicate the RNA-seq evidence for *HOXB-AS3* variant 3 as the predominant variant expressed in Mb. **(d)** RRBS data for two control Mb cell strains and Mt preparations derived from them, as well as two fetal lung fibroblast cell strains analyzed as technical duplicates. Arrows and highlighted subregions are described in the text.

**Figure 6:**
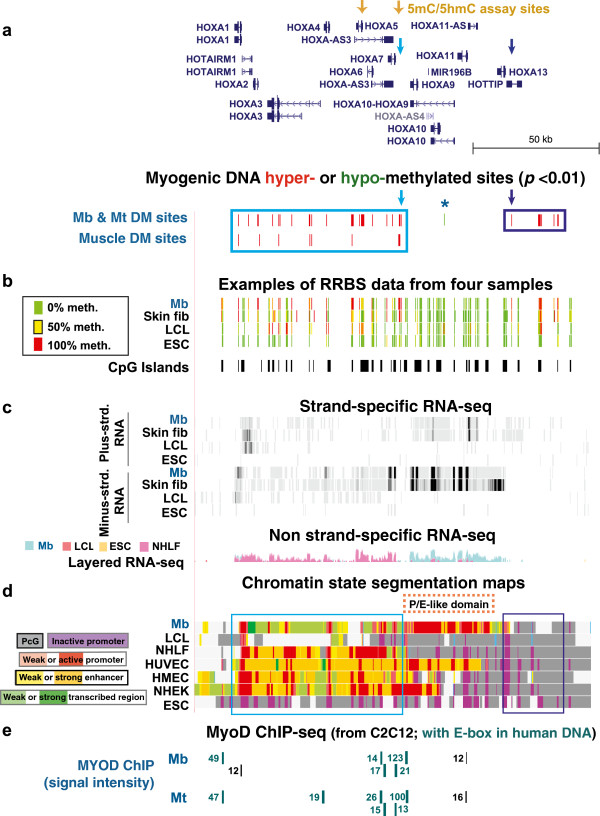
**Peripheral myogenic hypermethylation and a central myogenic hypomethylated site in the *HOXA *gene cluster. (a)** 187 MbMt-hypermethylated and 20 muscle-hypermethylated sites as well as one MbMt-hypomethylated site in the chr7:27,116,782–27,273,459 region. **(b)** Examples of RRBS data. **(c)** RNA-seq profiles as in Figure 1. **(d)** Chromatin state segmentation analysis. **(e)** MyoD binding sites from C2C12.

Not only was *HOXD4* silent in most of the examined cells types, including Mb, but this was also the case for the rest of the *HOXD* cluster, especially the 3′ half of the cluster (Figure 1c). Similarly, there was silencing-associated H3K27me3 throughout the gene cluster in Mb, Mt and most examined nonmyogenic cell types (Figure 1d, PcG, and Additional file 3) as determined by whole-genome chromatin immunoprecipitation/next-generation DNA sequencing (H3K27me3 ChIP-seq; ENCODE/Broad Institute, http://genome.ucsc.edu/). There was an unusually high concentration of CpG islands in the *HOXD* cluster and the other three *HOX* clusters (Figures 1, 2, 3, 4, 5 and 6), but this cannot explain the myogenic hypermethylation in *HOX* gene clusters. For example, there was a much higher density of MbMt-hypermethylated sites in the 3′ half of the *HOXD* gene cluster relative to the 5′ half, but not a higher density of CpG islands (Figure 1a).

An important question is raised by our finding of much myogenesis-associated DNA hypermethylation in the 3′ half of the *HOXD* cluster while H3K27me3 was seen throughout this region in most examined cell types, including Mb and Mt. Why did the many examined populations of myogenic cells display DNA hypermethylation in this multigene region compared with other cell types, even though the myogenic and nonmyogenic cells shared polycomb silencing which might have sufficed for repression of genes in this region [51]? Although regions of DNA methylation and H3K27me3 sometimes overlap, the relationships between these two epigenetic markers are varied and region-specific [52]. Our findings could be explained most easily by the hypothesis that, for the 3′ half of *HOXD*, polycomb group silencing at the chromatin level does not suffice for repression of 3′ *HOXD* genes in Mb and Mt, and, specifically in these cells, H3K27me3 needs to be supplemented with DNA methylation. Without the DNA hypermethylation, myogenic progenitor cells might be more susceptible to leaky expression of 3′ *HOXD* genes than are most other cell types. Alternatively, *HOXD*-encoded proteins or ncRNAs generated from the 3′ half of the cluster might be deleterious specifically to myogenic progenitor cells. Consistent with a combined role of DNA methylation and H3K27me3 in some types of *HOX*/*Hox* gene regulation, recently it was shown that experimentally induced DNA hypomethylation in mouse embryonic fibroblasts led to decreased H3K27me3 at *Hox* genes, including genes in the 3′ half of the *Hoxd* gene cluster [53]. Some *Hox* genes were shown to be derepressed upon DNA demethylation. Our study suggests that the roles of DNA methylation of *HOX* genes during development are more nuanced than can be seen in a study of one cell type because NHLF (IMR90), ESC and LCL samples exhibited much H3K27me3 in the 3′ half of the *HOXD* cluster despite very little DNA methylation there (Figures 1b and 1d, Additional file 3). In contrast, Mb and Mt displayed both H3K27me3 and much DNA methylation in this region.

### Myogenic hypermethylation in the *HOXC* cluster bordering an H3K4me3-rich multigenic region

MbMt-hypermethylation was also seen in the *HOXC* cluster (Figure 2a), but, unlike *HOXD* genes, many *HOXC* genes were moderately or strongly expressed in Mb and Mt but not in NHLF, LCL, ESC and HUVEC samples (ENCODE/RNA-seq, California Institute of Technology and Cold Spring Harbor Laboratory; Figure 2c and Additional file 1). Foreskin fibroblasts were the other examined cell type that displayed considerable expression of *HOXC* genes, although less than for Mb (Figure 2c, skin fib). Figure 2d shows a distillation of ChIP-seq chromatin epigenetic data (H3K4me1, 2 or 3; H3K27Ac; H3K9Ac; H3K27me3; H3K36me3; H4K20me1; and CTCF binding) by Ernst *et al*. using a multivariate hidden Markov model (ENCODE/Broad Institute; [54]) to predict chromatin states (color-coded chromatin state segmentation maps). In much of the central, multigenic region of the *HOXC* cluster in Mb and Mt, the chromatin state segmentation map shows chromatin with the features of a strong promoter, especially a strong signal for H3K4me3. The H3K4me3 was present in broad intragenic and intergenic subregions in Mb (Figure 2d, Mb, red subregions), as was found for transcribed *HOX* gene clusters in murine embryonic fibroblasts [30]. This active promoter-like (or active enhancer-like [55]) chromatin rich in H3K4me3 in the middle of the *HOXC* gene cluster was interspersed with a type of chromatin typically associated with active enhancers (H3K27Ac plus H3K4me1; Figure 2d, Mb, orange subregions). We refer to such a multigenic region consisting largely of chromatin with the typical characteristics of active promoters and enhancers as a P/E-like domain. The P/E-like domain probably reflects, in part, the high density of ncRNA genes, including undocumented ones, and alternative transcription start sites within *HOX* gene clusters [56-58]. This P/E domain is also predicted to contain a MYOD binding site (Figure 2e) because it contains a sequence which is orthologous to a genomic sequence in C2C12 mouse Mb and Mt samples that bound MyoD in MyoD-ChIP-seq profiles [59]. Moreover, this site in the human genome has a centrally located CAGCTG E-box, which is found in many MYOD/MyoD binding sites [59].

Within a 650-kb region centered over the approximately 130 kb *HOXC* cluster, Mb displayed a P/E-like domain only at the *HOXC* cluster, and this cluster was the most prominent gene region with myogenesis-associated gene expression (Figures 3c and 3d). Many strong C2C12-inferred MYOD sites were located outside the *HOXC* cluster (Figure 3a). We hypothesize that these may be part of long-distance, tissue-specific *HOX* enhancers, like those previously described [35], or may help organize long-range chromatin structure around the *HOXC* cluster. Moreover, as for all the *HOX* clusters, the *HOXC* region with its high concentration of CpG islands and RRBS-detected, MbMt-hypermethylated sites was surrounded by DNA that had a low density of both (Figure 3b).

Many of the MbMt-hypermethylated sites within the *HOXC* gene cluster (Figure 2a, blue boxes) surround the myogenesis-associated P/E-like domain. Their location relative to chromatin epigenetic marks suggests that they are part of a boundary element preventing the spread of the central P/E-like domain and the associated high levels of expression into the adjacent chromatin (Figures 2a and 2d and Additional file 4). This hypothesis would be consistent with observed negative relationships between DNA methylation and H3K4 methylation [60]. CTCF sites often function as boundary elements or insulators [61]. There were no strong CTCF sites at the 5′ end of the P/E-like domain in Mb, and there was only a constitutive CTCF site near the other end (Figure 2e, bottom, and Additional file 4).

The cluster of myogenic DM sites at the 3′ border of the P/E-like domain (Figure 2a, dark blue arrow) overlapped a CpG island in *HOXC4* variant 1 (intron 1) and *HOXC6* variant 2 (last exon; Figure 3a and ENCODE/RNA-seq, California Institute of Technology). These genes were both preferentially expressed in Mb and share the same TSS. *HOXC5* variant 2 shares this TSS, too, but it had no detectable transcripts according to RNA-seq data (ENCODE/California Institute of Technology and Cold Spring Harbor Laboratory). Differential splicing will help determine relative expression of these overlapping *HOXC4*, *HOXC5* and *HOXC6* genes. Because DNA methylation can affect the relative steady-state levels of RNA by modulating the rate of progression of the RNA polymerase II (RNA Pol II) complex in diverse ways [62,63], we hypothesize that the 41 intragenic MbMt-hypermethylated DNA sites in these three overlapping genes help regulate differential splicing in this region through effects on RNA Pol II elongation. Such effects of intragenic DNA methylation are likely to be gene-specific and/or cell type-specific [64,65].

### Myogenic hypermethylation downstream of *HOTAIR*

Near the 5′ end of the *HOXC* P/E-like domain in Mb, there were eight MbMt-hypermethylated sites in a CpG island approximately 1 kb downstream of *HOTAIR*, a long noncoding RNA (lncRNA) gene (Figure 2a, light blue arrow, and Additional file 4). Mb displayed a moderate level of expression of *HOTAIR*, whereas there was little or no expression in most of the other studied cell types, with the exception of foreskin fibroblasts (Figure 2c and Additional file 1), which were highly methylated in the *HOTAIR* downstream region like Mb and Mt (Figure 2b). Hypermethylation of *HOTAIR*’s 3′ downstream CpG island was also seen by Lu *et al*. [66] in breast cancer and correlated with expression. The MbMt hypermethylation downstream of *HOTAIR* included the 3′ half of *HOXC12* (Figure 2 and Additional file 4). Lu *et al*. proposed that one of the functions of *HOTAIR* downstream methylation was to facilitate transcription termination at *HOXC12*. They reported no good matches in this region to DNA poly(A) signals [66] as detected by a program for predicting optimal AATAAA poly(A) termination signals [67]. However, we found that the program indicated two individually low-rated poly(A) signals, 9 bp apart, 2.5 kb downstream from the 3′ end of the RefSeq *HOXC12* sequence. We noted that the sense transcript from *HOXC12* extends approximately to these two poly(A) signals downstream of the canonical RefSeq sequence (Additional file 4, RNA-seq, orange triangle).

We propose that DNA methylation in this *HOXC* subregion not only acts as part of a boundary element but also facilitates transcription termination through RNA Pol II pausing at *HOXC12.* This in turn may favor expression of the oppositely oriented *HOTAIR*. In a study of mouse *Hoxc6* and *Hoxc8* in embryonic fibroblasts, Tao *et al*. provided several lines of evidence for DNA methylation causing repression at the transcription elongation step due to long-lived pausing of RNA Pol II [63]. They showed that the effect of demethylation on *Hox* transcription was tissue-specific and specific to individual *Hox* genes [65]. Consistent with the results of Tao *et al*., the P/E-like domain in *HOXC* in Mb includes the *HOXC6* promoter region and *HOXC8*, both of which were mostly or completely unmethylated and were upregulated in Mb and Mt vs. other cell types (Figure 2, Additional file 1, and data not shown).

*HOTAIR* RNA in trans represses genes across the whole *HOXD* gene cluster by recruiting chromatin-modifying polycomb group proteins, which results in extensive H3K27 trimethylation of the *HOXD* cluster [68]. We hypothesize that the preferential expression of *HOTAIR* that we found in myogenic progenitor cells is partly responsible for their *HOXD* DNA hypermethylation. This would be consistent with the recent report that knockdown of *HOTAIR* caused a decrease in DNA methylation of the promoter region of the *PTEN* gene in laryngeal squamous carcinoma cells [69].

### Hypermethylation and transcription in a *HOXB* subregion of myogenic progenitor cells

The *HOXB* cluster, unlike the *HOXC* and *HOXD* clusters, displayed most of its MbMt hypermethylation in a subregion with considerable gene expression in myogenic progenitor cells, namely, the subregion containing *HOXB4*, *HOXB5*, *HOXB6* and *HOXB7*, and *HOXB-AS3* (Figures 4a–4c and Additional file 1). There was even higher expression of these genes in HUVECs or NHLFs. *HOXB-AS3* transcripts in Mb were mostly variant 3 (Figure 5c, pink boxes). There were 20 MbMt-hypermethylated sites from approximately 40 to 400 bp after the TSS of *HOXB-AS3* variant 3, which overlapped the single intron and last exon of *HOXB5* as well as a CpG island (Figures 4a and 5a, gray triangle). The higher levels of expression of *HOXB5* and several variant *HOXB-AS3* transcripts in NHLF vs. Mb were paralleled by a lack of methylation in this subregion in lung fibroblasts at sites that were hypermethylated in Mb and Mt (Figure 5d, purple arrows). The opposite DNA methylation pattern was seen at exon 2 in *HOXB-AS3* variants 1 and 4, where NHLF displayed DNA methylation, whereas Mb, Mt and most other cell types had little or no methylation (Figure 5, tan highlighting and data not shown). These findings could be explained most easily by the following hypothesis. DNA methylation in Mb close to the *HOXB-AS3* variant 3 TSS and further upstream may be downmodulating its transcription and suppressing transcription of the other *HOXB-AS3* variants in Mb, whereas DNA methylation at exon 2 in NHLF might control splicing of *HOXB-AS3* transcripts specifically in those cells. Moreover, the results from NHLF suggest that antisense *HOXB-AS3* transcription favors the sense *HOXB5* expression, as indicated by other studies of antisense vs. sense genes in *HOX* gene clusters [58,70]. *HOXB5* expression might be fine-tuned by the effects of differential methylation on the level of transcription of overlapping *HOXB-AS3* gene isoforms.

### Myogenic hypermethylated sites may serve as a boundary at the end of highly transcribed *HOXA* chromatin in myogenic cells

Like the *HOXC* cluster, the *HOXA* cluster displayed much hypermethylation on both sides of a P/E-like domain in Mb and Mt (Figures 6a and 6d, boxed regions). Genes in the *HOXA* P/E-like domain, including *HOXA9*, *HOXA10*, *HOXA11* and *HOXA11-AS*, were preferentially expressed in Mb and HUVEC vs. NHLF, an LCL, and ESC (ENCODE/RNA-seq, California Institute of Technology) and were expressed at higher levels in Mb than were genes bordering this domain, namely, *HOXA7* and *HOXA13* (Figure 6c and Additional files 1 and 5). Consistent with the findings for Mb and HUVEC, 5′ *Hoxa* genes are involved in the skeletal muscle and endothelial cell lineages during mouse embryo development and in the adult mouse [15,71]. Between *HOXA7* and *HOXA9* was a cluster of 15 MbMt-hypermethylated sites and four muscle-hypermethylated sites (Figure 6a, light blue arrow, and Additional file 5). Surrounding this subregion was the highest concentration of orthologous sites for MyoD binding in the *HOX* gene clusters (ChIP-seq profiles of C2C12 mouse Mb and Mt [59]), and all of these contained centrally located, MYOD/MyoD-like CAGCTG E-box sequences (Figure 6e and Additional file 5). We propose that the clusters of MbMt-hypermethylated sites here and at the other border of the P/E-like domain help establish the boundaries of this myogenesis-associated domain either alone or in conjunction with nearby constitutive CTCF binding sites (Additional file 5 and data not shown).

### Myogenic hypomethylation in *HOXA* and extensive undermethylation in ESC and several nonembryonic cell types

The only MbMt-hypomethylated site found in the *HOX* gene clusters was located in the middle of the MbMt-associated P/E-like domain of the *HOXA* cluster (Figure 6a and Additional file 5, asterisk). This site is 1.7 kb upstream of the protein-encoding isoform of *HOXA10* and inside the single intron of the ncRNA-encoding isoform of this gene. *Hoxa10* is implicated in limb muscle development and expressed in murine hindlimb progenitor muscle cells from neonatal muscle [72]. Strand-specific RNA-seq indicates that both the lncRNA and mRNA isoforms of *HOXA10* were expressed in Mb and HUVEC (Figure 6, and data not shown). The MbMt-hypomethylated site may be part of an extended myogenesis-associated enhancer for the *HOXA10* gene in the P/E-like domain.

One of the sample types with the least DNA methylation throughout the *HOX* clusters was ESC (Figures 1, 2, 4 and 6). Moreover, *HOX* clusters in ESC had less DNA methylation than fibroblasts and monocytes [41]. This exceptional lack of *HOX* DNA methylation was also seen for astrocytes, choroid plexus epithelial cells, iris pigment epithelial cells and retinal pigment epithelial cells (data not shown). The similar *HOX* DNA epigenetics of these four cell types is probably due to their common derivation from the neuroectoderm.

### Similarities and differences in methylation of paralogous *HOX* genes and comparison of Mb and ESC epigenetic marks

*HOX* clusters provide the opportunity to compare the epigenetics of paralogous sets of genes. Paralog group 4 *HOX* genes all had RRBS data. Of these genes, *HOXA4*, *HOXB4* and *HOXD4* had MbMt-hypermethylated sites in the coding sequences of the last exon (Additional file 1), which encodes the homeodomain. *HOXC4* was also methylated in this subregion in Mb and Mt, as were a number of other types of cell cultures, so that this subregion was not scored as hypermethylated (data not shown). Four other *HOX* genes also had clusters of hypermethylated sites in the coding sequences of the last exon (Additional file 1).

*HOX* gene myogenic hypermethylation was also found in gene subregions without much sequence similarity. This includes the 3′-untranslated region (3′-UTR) of *HOXB6* and *HOXC5*, exon 1 of *HOXA6*, the 2-kb upstream region of *HOXC12* and an internal exon (exon 3 of four exons) of *HOXA3* (Additional file 1). All of these genes had mostly unmethylated CpGs detected by RRBS in Mb and Mt in their vicinity, so that the detected MbMt hypermethylation was not just due to large, continuous blocks of DNA methylation. *HOXA6* and *HOXC6*, both of which had two exons, illustrate variety in the DNA methylation of paralogs. They exhibited, respectively, hypermethylation (and gene silencing) and little or no methylation in their first exon (and moderate gene expression) in myogenic progenitor cells (Additional file 1).

We found that subregions of H3K4me2 in ESCs often were located at MbMt-hypermethylated sites (Additional files 3, 4, 5 and 6, purple triangles). H3K4me2 marks (transcription promoting) in ESCs often overlap H3K27me3 signals (transcription repressing) and hence are called *bivalent chromatin subregions* that are paused for activity [17]. We hypothesize that the frequent overlap of ESC H3K4me2 with MbMt hypermethylation is due to the resolution of a bivalent chromatin mark to a univalent H3K27me3 mark with the addition of *de novo* DNA methylation early in the differentiation of the skeletal muscle lineage.

### Unusually high 5hmC levels at a hypermethylated site in *HOXB5* in skeletal muscle

Because Mb and Mt have particularly high levels of the RNA encoding TET1 and TET2, enzymes that generate 5hmC from 5mC residues [11], and RRBS cannot distinguish between 5hmC and 5mC [42], it was important to determine relative amounts of these modified C residues at representative *HOX* cluster sites. We quantified 5mC and 5hmC at a MbMt-hypermethylated *Msp*I site (5′-CCGG-3′) in the single introns of *HOXB5* and *HOXD4*, exon 1 of *HOXA5*, exon 2 of *HOXC6*, and 1.7 kb upstream of the TSS of *HOXA7* (Figures 1, 2, 4 and 6) by an enzymatic assay that involves glucosylation of 5hmC by T4 phage β-glucosyltransferase (β-GT; Epimark; New England Biolabs, Ipswich, MA, USA), digestion with *Msp*I or *Hpa*II and real-time PCR [11]. Using sets of samples independent from those for RRBS, the hypermethylation of these five sites in Mb and of the *HOXD4* and *HOXA7* sites in skeletal muscle was verified by this assay (Table 1). Moreover, we found that all or almost all of the hypermethylation at these sites in Mb was due to 5mC rather than to 5hmC.

**Table 1:** **Quantification of 5mC and 5hmC at five tested CCGG sites in the four *HOX *clusters**

**Location of tested site**	**(5mC + 5hmC)/All C (ratio)**^**a**^										
	***5hmC/(5mC + 5hmC) (%)***										
	**Mb1**	**Mb2**	**Skel muscle1**	**Skel muscle2**	**Heart1**	**Heart2**	**Brain1**	**Brain2**	**Leuk1**	**Leuk2**	**Fib**
*HOXB5* in1	**0.93**^**b**^	**0.80**	**0.43**	**0.41**	0.06	0.01	0.04	0	0.05	0.00	0.03
	*0%*	*15%*	***63%***^***c***^	***100%***	***100%***	***100%***	***100%***		*0%*	*0%*	***100%***
*HOXA5* ex1	**0.99**	**0.96**	0.19	0.04	0.05	0	0.02	0.01	**0.97**	**0.86**	0.02
	*0%*	*1%*	*17%*	*0*	*0*		***100%***	***62%***	*0%*	*0%*	*0%*
*HOXA7* upstr	**0.99**	**0.65**	**0.55**	**0.48**	0.01	0	0.02	0.01	0.07	0.02	0.02
	*0%*	*1%*	*15%*	*0%*	*0%*		***78%***	***62%***	***71%***	*0%*	*4%*
*HOXC6* ex2	**0.98**	**0.46**	0.26	0.01	0.01	0	0.04	0	0.18	0.03	0.02
	*0%*	*2%*	*31%*	*0%*	*0%*		***100%***		*0%*	*0%*	*1%*
*HOXD4* in1	**0.98**	**0.92**	**0.78**	**0.54**	0.08	0.03	0.05	0	0.08	0.01	1.00
	*0%*	*0%*	*10%*	*0%*	***48%***	*0%*	***100%***		*0%*	*0%*	*3%*

^a^5hmC and 5mC were quantified by an enzymatic assay (Epimark; New England Biolabs) coupled with real-time PCR as described in Methods. Two biological replicates each were tested for myoblasts, skeletal muscle biopsy samples, heart and brain autopsy samples, and leukocytes. One sample of foreskin fibroblasts was assayed. The tested positions of CpG sites (in CCGG sequences) were as follows (hg19): *HOXB5* intron 1, chr17:46670028; *HOXA5* exon 1, chr7:27183175; *HOXA7* upstream, chr7: 27197926; *HOXC6* upstream, chr12:54423458; and *HOXD4* intron 1, chr2:177017078. These positions are also indicated in Figures 1, 2, 4 and 6.

^b^Samples with ratios of (5mC + 5hmC)/total C greater than 0.4 are shown in in boldface.

^c^Samples with more than 40% of modified C as 5hmC are shown in boldface.

Surprisingly, only the skeletal muscle samples at the assayed CpG site in *HOXB5* intron 1 displayed considerable levels of 5hmC (27% or 41% of all C as 5hmC), and, remarkably, these samples exhibited more 5hmC than 5mC (5hmC and no 5mC or mostly 5hmC; Table 1). In a previous study of genomic DNA 5hmC mapping in mouse embryonic stem cells (E14) ([73] and unpublished data), only about 2% of the mapped 5hmC sites were found to contain higher levels of 5hmC compared to 5mC using *Msp*I and *Hpa*II differential digestion after β-glucosylation, as in this study. At the *HOXB5* site analyzed in the present study, all the detected modified C was 5hmC in the heart samples, one of the two assayed cerebellum samples and the foreskin fibroblast sample. However, the overall levels of modified C in these samples were much lower than in skeletal muscle: only 1% to 6% vs. 41% to 43%, respectively (Table 1). In a study of the *HOXA* gene cluster in NT2 embryonal carcinoma cells before and after retinoic acid-induced differentiation, Bocker *et al*. [45] found that gene activation was accompanied by conversion of much 5mC to 5hmC. Their analysis involved immunoprecipitation using antibodies to 5hmC or 5mC, which does not allow comparisons of relative amounts of 5hmC to 5mC. Our results also indicate that some *HOX* genes can have more genomic 5hmC in differentiation products than in precursor cells, although, in this case, the comparison is adult tissue to progenitor cells. This finding is also consistent with our previous demonstration that skeletal muscle had twice the average genomic 5hmC content of Mb or Mt in an assay of overall levels of genomic 5hmC [11].

## Conclusions

Our profiling of differential DNA methylation in *HOX* gene clusters suggests that myogenesis-associated hypermethylation plays diverse roles in controlling cell type-specific expression of *HOX* genes and does not simply mirror chromatin epigenetics. Specific roles for developmentally associated, differential methylation of *HOX* gene regions would be consistent with the unusually high density of the sense and antisense genes, alternative promoters and alternative transcription termination sites in *HOX* gene clusters [56-58] and the need for tight control of expression of these key developmental regulatory genes. For example, we found extensive DNA hypermethylation in the 3′ half of *HOXD* selectively in myogenic cells and skeletal muscle tissue, whereas H3K27me3 was present throughout the *HOXD* gene cluster in many cell types, including Mb and Mt. This finding is consistent with the hypothesis that the skeletal muscle lineage needs especially tight or stable silencing of transcription of the *HOXD* gene cluster conferred by DNA methylation plus polycomb silencing. Moreover, our results indicate that myogenic DNA hypermethylation was often localized to bivalent ESC subregions, which may have been resolved to stably repressed subregions by *de novo* DNA methylation during differentiation. This is similar to a model for DNA hypermethylation of polycomb protein-controlled genes in cancer [74,75]. At the *HOXA* and *HOXC* gene clusters, the pattern of tissue-specific epigenetic marks suggests another function of myogenic DNA hypermethylation. In these gene clusters, subregions rich in myogenic DNA hypermethylation appear to be part of boundaries around a central multigenic region consisting of mostly enhancer- or promoter-type histone modifications. This DNA hypermethylation might help prevent the spreading of activating histone modifications from the central region of *HOXA* and *HOXC* gene clusters to their periphery.

Our study also suggests that myogenic hypermethylation of DNA might partly downregulate in cis the level of transcription of some *HOX* antisense ncRNA genes that positively control expression of overlapping protein-encoding *HOX* genes, such as *HOXB-AS3* and *HOXB*5. Myogenic hypermethylation from intragenic or intergenic locations could exert its effects on enhancers by decreasing transcription of the enhancer itself as well as by repression of canonical promoters of protein-encoding or lncRNA genes [76,77]. Moreover, our results are consistent with the hypotheses that hypermethylation within gene bodies affects which RNA isoforms are generated by modulating differential splicing and the use of alternate promoters [62,76,78,79].

Yet other relationships between differential methylation and transcription were indicated by the association of upregulation of *HOTAIR* in the *HOXC* cluster and hypermethylation of *HOTAIR*’s immediate downstream sequences in myogenic progenitor cells and foreskin fibroblasts. In addition, the one example of myogenic hypomethylation in the *HOX* gene clusters was in the single intron of *HOXA10* in a region with the chromatin features of a myogenesis-associated enhancer. This tissue-specific DNA hypomethylation might activate or help maintain the activity of a tissue-specific enhancer, consistent with the positive association of DNA hypomethylation and inducible enhancers [80]. The dynamic nature of developmentally linked changes in DNA was evidenced by our finding that, at five tested representative CpG sites displaying myogenic hypermethylation, the levels of 5hmC were low or negligible in the skeletal muscle lineage, with the prominent exception of an intronic region in *HOXB5* in skeletal muscle tissue but not in muscle progenitor cells. In summary, our study of myogenesis-associated differences in DNA methylation indicates the importance of considering a wide variety of possible roles for differential DNA methylation when studying disease-linked epigenetic changes.

## Methods

### Samples

All the Mb cell strains used for methylation analysis were propagated from muscle biopsy samples that were previously described [11]. Mt samples were obtained from these myoblast cell strains by serum limitation for 5 days [11]. By immunostaining [81], we demonstrated that all batches of myoblasts contained more than 90% desmin-positive cells and that myotube preparations had more than 75% of their nuclei in multinucleated, desmin-positive and myosin heavy chain-positive cells. Four of the nine Mb and Mt samples were Mb-Mt pairs from two normal controls, and five were from two facioscapulohumeral muscular dystrophy patients or an inclusion body myositis patient; however, all Mb and Mt samples predominantly shared the same myogenesis-associated epigenetic marks [11]. The other cell cultures for DNA methylation profiling and assessment of differential methylation in myogenic vs. nonmyogenic cell cultures and the tissue samples and the two primary (not passaged) cell cultures (hepatocytes and pancreatic islets) used for DNA methylation profiling to identify skeletal muscle-associated differential methylation were previously described normal samples [11]. All cell cultures were untransformed, except for the LCLs, which had been transformed *in vitro* by Epstein-Barr virus. Three control Mb or Mt samples were used for the combined data shown in DNase-seq profiles (Additional files 3, 4, 5 and 6). Two of these Mb samples and two of the Mt samples were from the same batches of cells used for RRBS. Although different sources of Mb and Mt were used for ChIP-seq, peaks of myogenesis-associated H3K4me3 or H3K4me2 usually overlapped DNase-seq peaks for these cell types (Additional files 3, 4, 5 and 6), as expected.

### DNA methylation profiling and statistical analyses

For the methylation analysis, high-molecular-weight DNA was extracted, digested with *Msp*I and used for bisulfite-based RRBS, including next-generation sequencing on an Illumina platform (Illumina Inc, San Diego, CA, USA) as previously described using the same samples as we used for our last study of myogenic differential methylation [11]. DNA methylation data for cell cultures and tissues are from the ENCODE project and available from the UCSC Genome Browser (http://genome.ucsc.edu/cgi-bin/hgTrackUi?hgsid=292099017&c=chr1&g=wgEncodeHaibMethylRrbs). BED files containing DNA methylation data for 5 Mb cell strains, 5 Mt samples (including one technical duplicate) and 47 nonmyogenic samples representing 23 unique cell lines from a variety of tissue types were aggregated into a single matrix. Rows were included for each site detected in any sample and used for assessing statistically significant differential methylation in the set of Mb plus Mt vs. non-muscle-cell cultures or skeletal muscle vs. non-muscle-cell cultures as previously described [11]. To increase the specificity of our analyses, we restricted our attention to those sites for which a change in methylation percentage of at least 50% was observed at a significance level of 0.01 or below. The closestBed program, (http://bedtools.readthedocs.org/en/latest/), a member of the bedtools suite [82], was used to map each DM site to the nearest gene using both protein-coding (NM*) and noncoding (NR*) genes and one isoform per gene as described previously [11].

### Chromatin epigenetic and transcription profiling

Data sets and sample information for histone modifications and CTCF binding, non-strand-specific RNA-seq and strand-specific RNA-seq were obtained from the ENCODE project (http://genomes.ucsc.edu/) via the laboratories of Bradley Bernstein (Broad Institute), Barbara Wold (California Institute of Technology) and Tom Gingeras (Cold Spring Harbor Laboratory), respectively. RNA-seq data were available for Mb, but not for Mt. RNA isoform analysis and quantification were done with the CuffDiff tool [83] using the above-mentioned ENCODE non-strand-specific RNA-seq database. For cell cultures that were represented in both RNA-seq profiles (Mb, HUVEC, GM12878, NHEK, NHLF and H1 ESC), the relative expression of different cell types was similar. Mb and Mt samples for ENCODE histone modification and CTCF profiling and Mb for RNA-seq were commercially obtained, and no immunostaining was described for them. For DNaseI hypersensitivity profiling, intact nuclei were treated with DNaseI and the DNaseI-hypersensitive fraction was analyzed by next-generation sequencing as previously described ([11,84]; ENCODE (http://genome.ucsc.edu/cgi-bin/hgTrackUi?hgsid=292099619&c=chr20&g=wgEncodeOpenChromDnase). In addition, we mined data from our previous expression profiling of Mb and Mt vs. 19 types of non-muscle-cell cultures on microarrays ([81]; GeneChip Exon 1.0 ST Array; Affymetrix, Santa Clara, CA, USA). There was overlap of three of the five control Mb or Mt samples for RRBS with the samples used for expression microarray profiling.

### Quantification of 5hmC and 5mC by enzymatic assay

For analysis of the levels of 5hmC and 5mC at a given *Msp*I site, we used an assay involving β-GT (Epimark; New England Biolabs). After incubation or a mock enzyme incubation, aliquots were digested with *Msp*I, which can cleave CCGG sites whether or not they are methylated or hydroxymethylated at the internal C residue, but not if they contain glucosylated 5hmC. In parallel, digestions were done with *Hpa*II, which can cleave CCGG sites only if they are unmodified at the internal C, and other aliquots were used as uncut controls according to the manufacturer’s instructions. Next, real-time PCR was performed and methylation status was calculated by subtraction of Ct values. The respective forward and reverse primers for PCR were as follows (5′ to 3′): *HOXC6*, ATCTTTAGGGGTCGGCTACG and CGCGTTAGGTAGCGATTGA; *HOXB5*, AGATGCCCACATTCAAGCTC and CAAGGGTGAGGCACTAGGAG; *HOXA7* upstream, GGTGTGGAGTGAGGGACAAC and CGATGCGACTGGGATTATTT; *HOXA5,* TTGCTCGCTCACGGAACTAT and TATAGACGCACAAACGACCG; and *HOXD4*, GGGATTTCCAAAATGCTTGA and ACCTCCTCAAACACACCCAC.

## Abbreviations

3′-UTR: 3′-untranslated region; 5hmC: 5-hydroxymethylcytosine; 5mC: 5-methylcytosine; ChIP-seq: Chromatin immunoprecipitation/next-generation DNA sequencing; DM: Differentially methylated; ESC: H1 embryonic stem cell; Fib2: Foreskin fibroblasts; GM12878: Lymphoblastoid cell line produced from blood by Epstein-Barr virus transformation; β-GT: β-glucosyltransferase; H3K27Ac: Histone H3 lysine 27 acetylation; H3K27me3: histone H3 lysine 27 trimethylation; H3K4me1: Histone H3 lysine 4 monomethylation; H3K4me3: Histone H3 lysine 4 trimethylation; HMEC: Human mammary epithelial cell; HUVEC: Human umbilical cord endothelial cells; LCL: Lymphoblastoid cell line; lncRNA: long noncoding RNA; Mb: myoblast; MbMt: Set of myoblasts plus myotubes; Mt: Myotube; ncRNA: Noncoding RNA; NHEK: Normal human epidermal keratinocyte; NHLF: Normal human lung fibroblast; PcG: Polycomb group; P/E-like domain: Promoter- and enhancer-type chromatin in a multigenic region; RNA Pol II: RNA polymerase II; RRBS: Reduced representation bisulfite sequencing; SA epith: Small airway epithelial cell; TSS: transcription start site.

## Competing interests

The authors declare that they have no competing interests.

## Authors’ contributions

KT prepared and immunocytochemically evaluated the Mb and Mt samples and helped with the Cufflinks analysis. CB and ML did the statistical analyses. JT, ZS and SP were responsible for the 5hmC and 5mC quantification. SC and CR helped with the bioinformatics analyses. LS and GEC did the DNaseI hypersensitivity profiling. LS did the liftover of C2C12 MyoD coordinates to the human genome. ME did bioinformatics analyses and wrote the manuscript. All authors read and approved the final manuscript.

## Supplementary Material

Additional file 1: Table S1 Correlations between myogenic differential methylation and transcriptional up- or downregulation in myogenic vs. nonmyogenic cells. Click here for file

Additional file 2: Figure S1 Myogenesis-associated DNA hypermethylation in the silenced *HOXD4* region. Click here for file

Additional file 3: Figure S2 Myogenic DNA hypermethylation and chromatin epigenetic marks in the *HOXD1*-to-*MIR10B* subregion of the *HOXD* gene cluster. Click here for file

Additional file 4: Figure S3 Myogenic DNA hypermethylation and chromatin epigenetic marks in the *HOXC-AS5*-to-*HOXC11* subregion. Click here for file

Additional file 5: Figure S4 Myogenic DNA hyper- and hypo-methylation and chromatin epigenetic marks in the *HOXA-AS3*-to-*HOXA11* subregion. Click here for file

Additional file 6: Figure S5 Myogenic DNA hypermethylation and chromatin epigenetic marks in the *HOXB4*-to-*HOXB9* subregion. Click here for file
