# Supplementary material for: DNA methylation and differentiation: HOX genes in muscle cells
Source: Epigenetics Chromatin. 2013 Aug 2;6:25. doi: 10.1186/1756-8935-6-25 (PMC3750649; doi:10.1186/1756-8935-6-25)
Supplement: Additional file 2: Figure S1 — Myogenesis-associated DNA hypermethylation in the silenced HOXD4 region. [file 1756-8935-6-25-S2.docx]

**Additional file 2, Figure S1. Myogenesis-associated DNA hypermethylation in the silenced *HOXD4* region.**

**
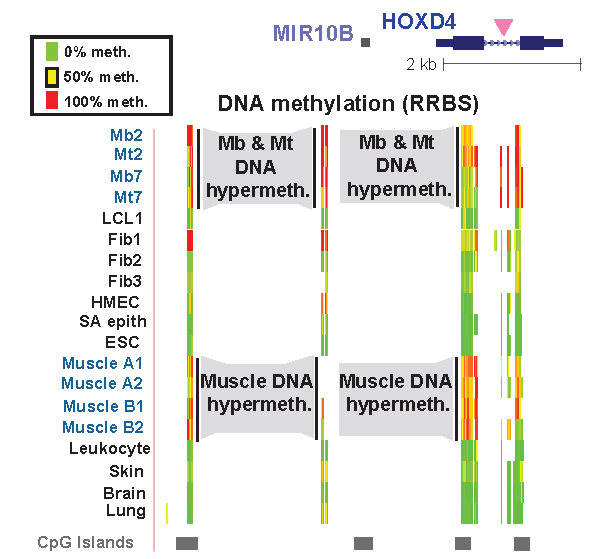
**

Each row of RRBS data comes from a different sample as indicated and was derived from the UCSC Genome Browser (<http://genome.ucsc.edu>; ENCODE/HudsonAlpha). This figure shows chr2:177,012,101-177,018,129 in the hg19 version of the human genome. The top part of the figure depicts the position of the *MIR10B* ncRNA gene and the *HOXD4* gene; exons are very thick lines for translated portions; moderately thick lines for untranslated 5’ and 3’ regions; and the intron is a thin line with the arrows indicating the direction of transcription. All tracks in this and other figures are aligned. The tracks for RRBS-determined CpG methylation (≥5 fragments per CpG site) are shown for the following samples: myoblast cell cultures from two individuals and myotubes derived from them (Mb and Mt), a lymphoblastoid cell line (LCL), skin fibroblasts from a girl (Fib1), neonatal foreskin fibroblasts (Fib2 and Fib3), human mammary epithelial cells (HMEC), small airway epithelial cells (SA epith), H1 embryonal stem cells (ESC), skeletal muscle samples from two individuals each analyzed as technical duplicates (Muscle), leukocytes, skin, brain, and lung. These samples are a subset of the ones used for the RRBS analysis in this study and were previously described in detail [1]. The bottom track displays the position of CpG islands relative to the RRBS-detected CpG sites. The pink triangle denotes a postulated internal promoter mentioned in the text. The significantly differentially methylated sites in this region are shown in Figure 1 of the main text (the tan highlighted subregion of that figure).

**Reference**

1. Tsumagari K, Baribault C, Terragni J, Varley KE, Gertz J, Pradhan S, Baddoo M, Crain CM, Song L, Crawford GE, et al: **Early de novo DNA methylation and prolonged demethylation in the muscle lineage**. *Epigenetics* 2013, **8**:317-32.
