# Supplementary material for: DNA methylation and differentiation: HOX genes in muscle cells
Source: Epigenetics Chromatin. 2013 Aug 2;6:25. doi: 10.1186/1756-8935-6-25 (PMC3750649; doi:10.1186/1756-8935-6-25)
Supplement: Additional file 3: Figure S2 — Myogenic DNA hypermethylation and chromatin epigenetic marks in the HOXD1-to-MIR10B subregion of the HOXD gene cluster. [file 1756-8935-6-25-S3.docx]

**Additional file 3, Figure S2. Myogenic DNA hypermethylation and chromatin epigenetic marks in the *HOXD1*-to-*MIR10B* subregion of the *HOXD* gene cluster.**

**
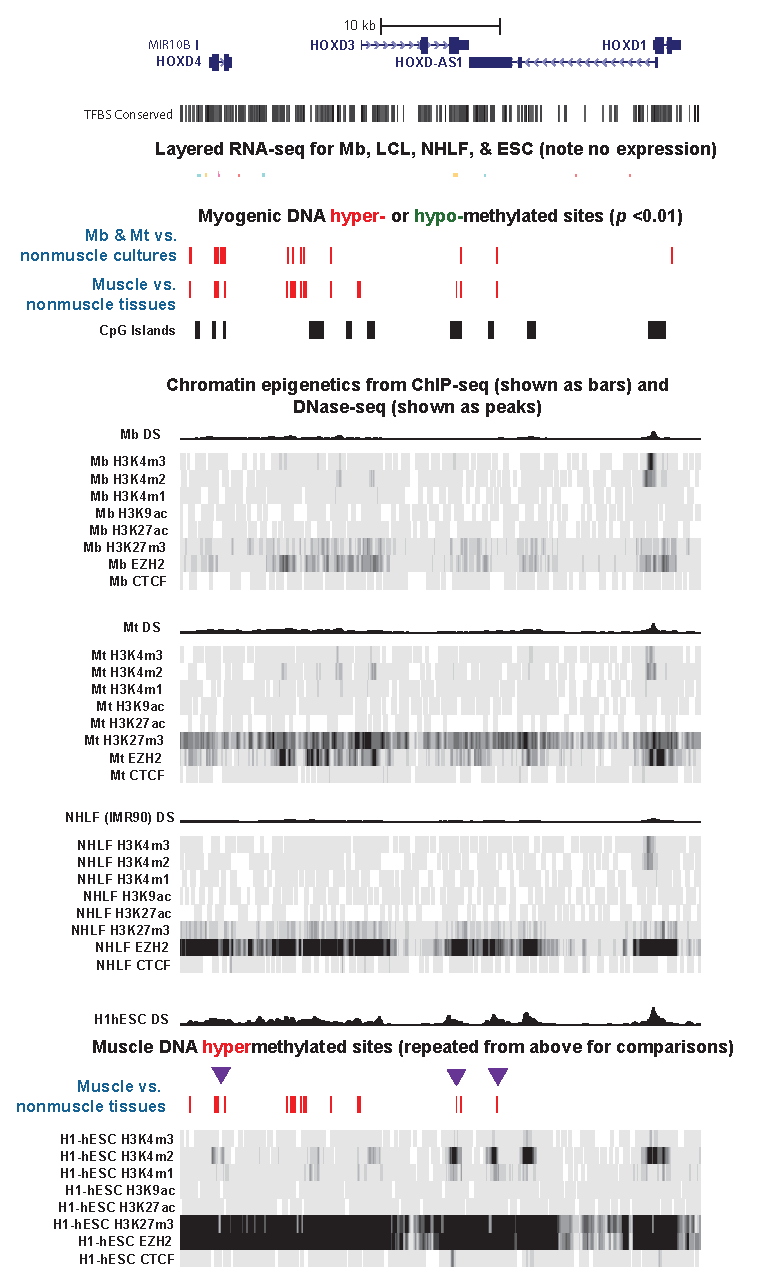
**

This figure shows a subregion of *HOXD* (chr2:177,013,670-177,057,252) that contained 34 CpG sites with significant hypermethylation in myoblasts (Mb) and myotubes (Mt) vs. nonmuscle cell cultures and 54 CpG sites with significant hypermethylation in skeletal muscle vs. nonmuscle tissue samples. In addition, the following tracks from ENCODE data at the UCSC Genome Browser (<http://genome.uscs.edu>) are displayed: TFBS, consensus sequence binding sites conserved between humans, mice, and rats; CpG islands; RNA-seq (CalTech); DS, DNaseI hypersensitive peaks by DNase-seq (Duke); histone modifications, polycomb group protein EZH2 binding by ChIP-seq, and CTCF binding by ChIP-seq (Broad). NHLF (normal human lung fibroblasts, IMR90 and AG4450, both fetal lung fibroblasts) were mostly unmethylated at RRBS-detected sites throughout this region, like the ESC cells in Figure 1. The NHLF sample used for modified histone ChIP-seq and for RNA-seq (ENCODE/CalTech, non strand-specific RNA-seq in Figures 1 , 2, 4 and 6) was adult-derived lung fibroblasts. The triangles denote MbMt-hypermethylated sites overlapping ESC-associated H3K4me2 or H3K4me3 signals from ChIP-seq, as described in **Results and discussion**.
