# Supplementary material for: DNA methylation and differentiation: HOX genes in muscle cells
Source: Epigenetics Chromatin. 2013 Aug 2;6:25. doi: 10.1186/1756-8935-6-25 (PMC3750649; doi:10.1186/1756-8935-6-25)
Supplement: Additional file 4: Figure S3 — Myogenic DNA hypermethylation and chromatin epigenetic marks in the HOXC-AS5-to-HOXC11 subregion. [file 1756-8935-6-25-S4.docx]

**Additional file 4, Figure S3. Myogenic DNA hypermethylation and chromatin epigenetic marks in the *HOXC-AS5*-to-*HOXC11* subregion.**

**
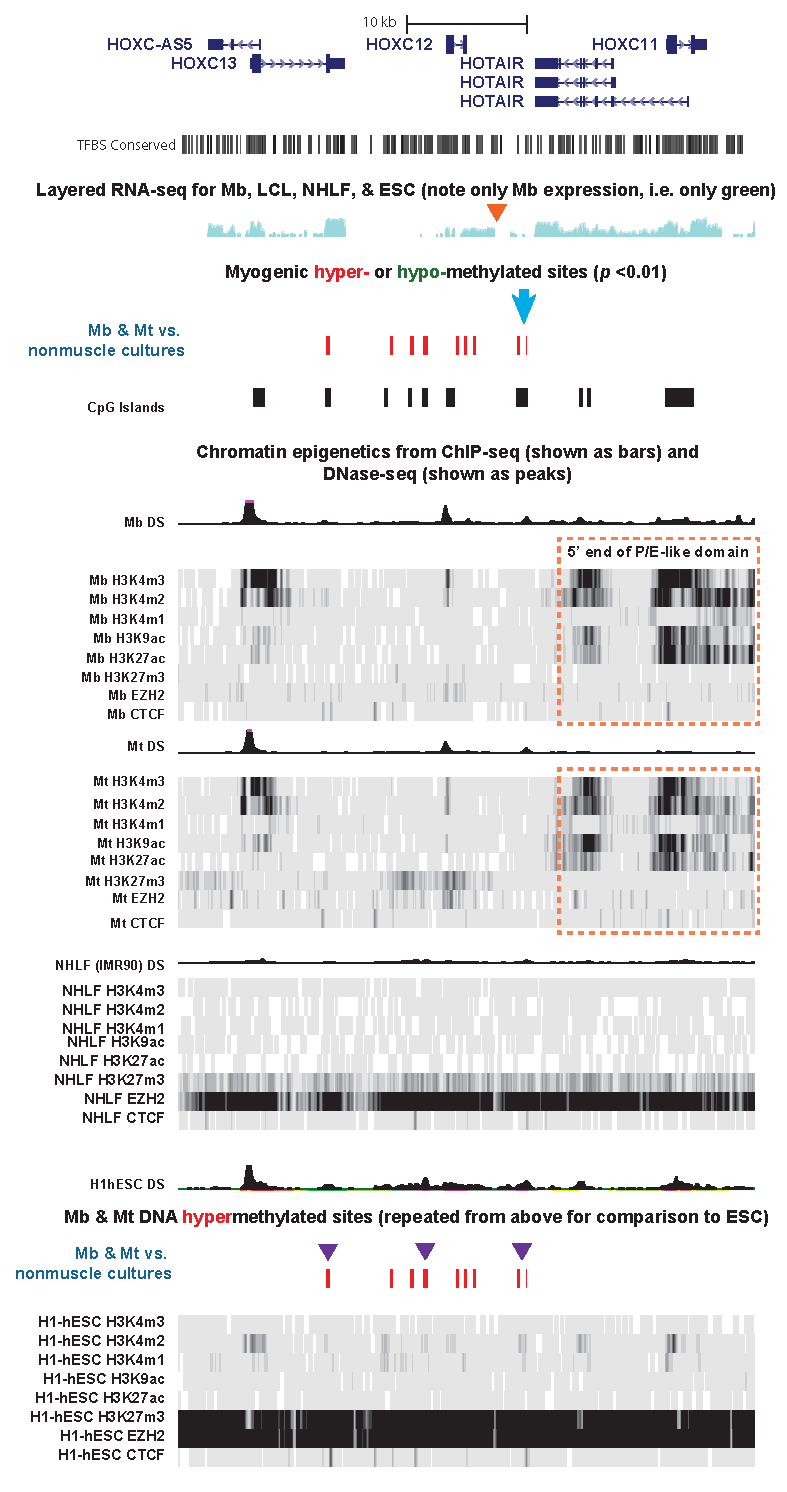
**

This figure shows a subregion of *HOXC* (chr12:54,326,577-54,374,216) that contained 68 CpG sites with significant hypermethylation in the set of Mb and Mt vs. nonmuscle cell cultures. Tracks from ENCODE data at the UCSC genome browser (<http://genome.uscs.edu>) are displayed as for Additional file 3 with the addition of the dotted orange box indicating the 5’ end of a P/E-like domain, a multigenic region consisting mostly of strong H3K4me3 signal (typical of active promoters) or H3K4me1 plus H3K27Ac signal (typical for active enhancers). Note the lack of strong CTCF sites near the 5’ end of the P/E-like domain in all cell types. The purple triangles indicate MbMt-hypermethylated sites overlapping ESC-associated H3K4me2 or H3K4me3 signals from ChIP-seq. The light blue arrow denotes the hypermethylated 5’ border of the P/E-like region as in Figure 2.
