# Supplementary material for: DNA methylation and differentiation: HOX genes in muscle cells
Source: Epigenetics Chromatin. 2013 Aug 2;6:25. doi: 10.1186/1756-8935-6-25 (PMC3750649; doi:10.1186/1756-8935-6-25)
Supplement: Additional file 5: Figure S4 — Myogenic DNA hyper- and hypo-methylation and chromatin epigenetic marks in the HOXA-AS3-to-HOXA11 subregion. [file 1756-8935-6-25-S5.docx]

**­­Additional file 5, Figure S5. Myogenic DNA hyper- and hypo-methylation and chromatin epigenetic marks in the *HOXA-AS3*-to-*HOXA11* subregion.**

**
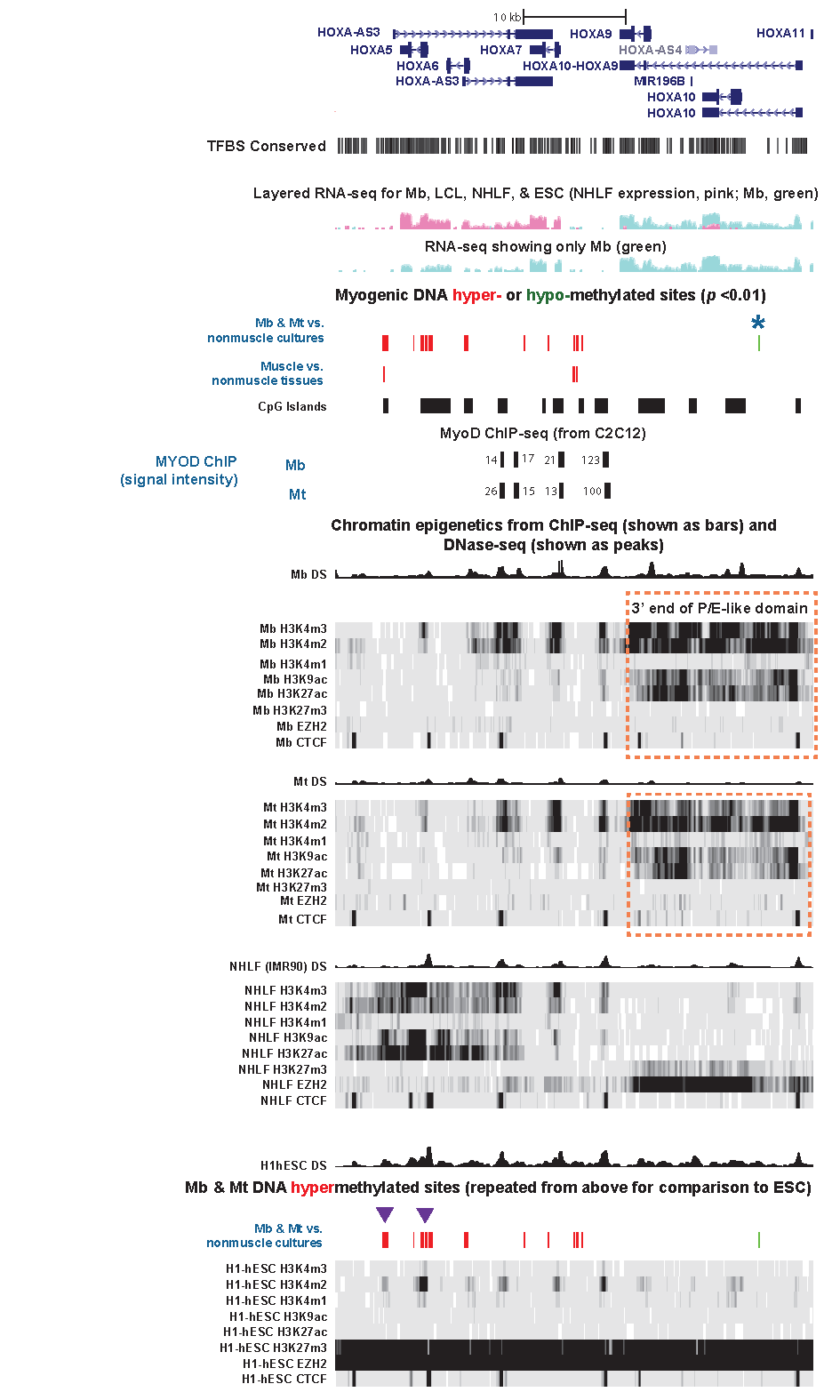
**

This figure shows a subregion of *HOXA* (chr7:27,174,343-27,220,977) that contained 89 CpG sites with significant hypermethylation in the set of Mb and Mt vs. nonmuscle cell cultures, five with significant hypermethylation in skeletal muscle vs. nonmuscle tissues, and one with hypomethylation in the MbMt set vs. nonmuscle cell cultures. Tracks from ENCODE data at the UCSC genome browser (<http://genome.uscs.edu>) are displayed as for Additional file 5. The purple triangles denote MbMt-hypermethylated sites overlapping ESC-associated H3K4me2 or H3K4me3 signals from ChIP-seq. Note the constitutive CTCF sites in this region.
