# Supplementary material for: DNA methylation and differentiation: HOX genes in muscle cells
Source: Epigenetics Chromatin. 2013 Aug 2;6:25. doi: 10.1186/1756-8935-6-25 (PMC3750649; doi:10.1186/1756-8935-6-25)
Supplement: Additional file 6: Figure S5 — Myogenic DNA hypermethylation and chromatin epigenetic marks in the HOXB4-to-HOXB9 subregion. [file 1756-8935-6-25-S6.docx]

**Additional file 6, Figure S4. Myogenic DNA hypermethylation and chromatin epigenetic marks in the *HOXB4*-to-*HOXB9* subregion.**

**
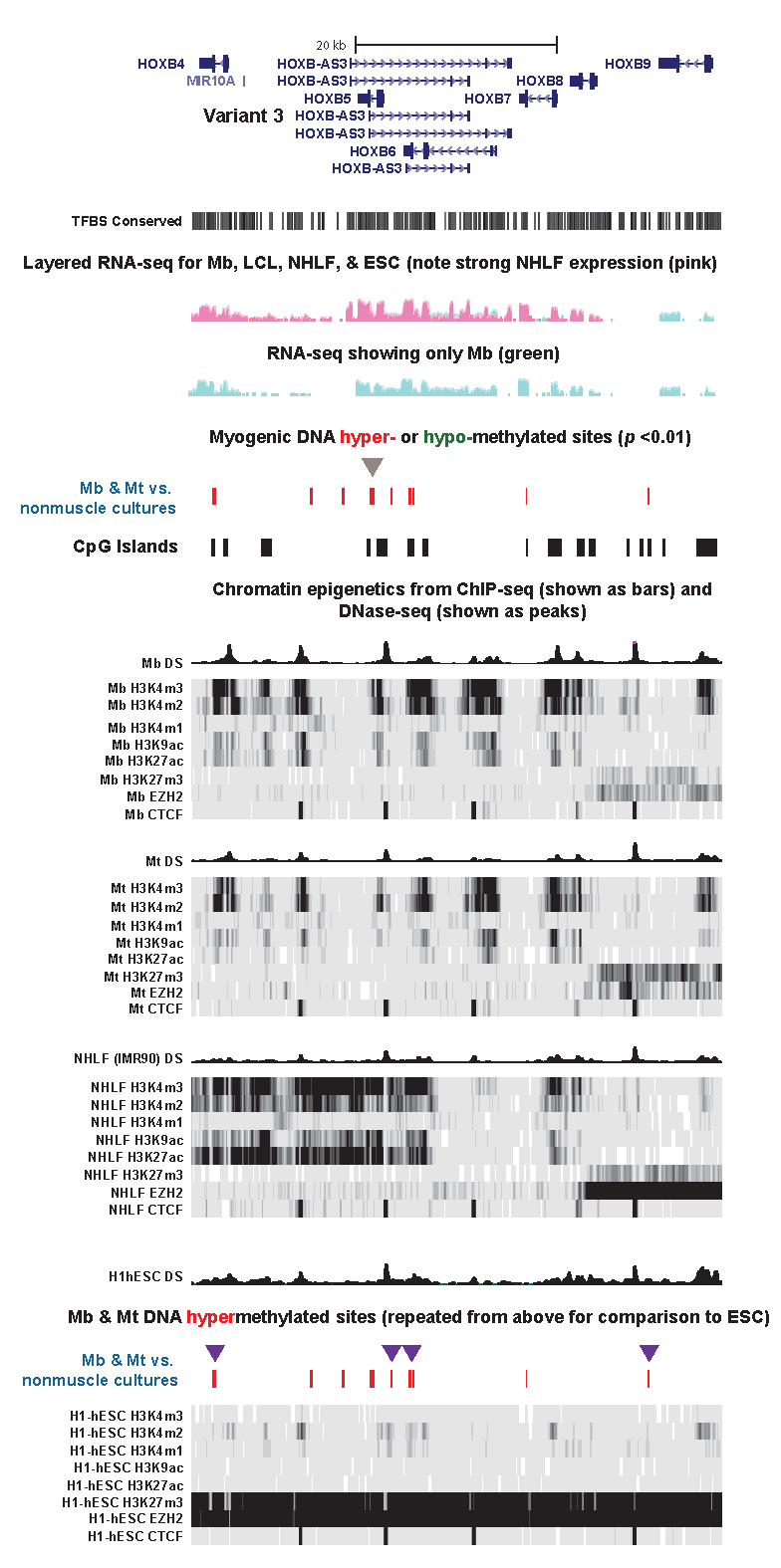
**

This figure shows a subregion of *HOXB* (chr17:46,652,025-46,704,727) that contained 59 CpG sites with significant hypermethylation in the set of Mb and Mt vs. nonmuscle cell cultures. Tracks from ENCODE data at the UCSC genome browser (<http://genome.uscs.edu>) are displayed as for Additional file 3 except that for clarity, we exhibit the RNA-seq profile from Mb alone as well as the overlaid profiles from Mb, LCL, NHLF, and ESC samples (ENCODE/CalTech). The purple triangles denote MbMt-hypermethylated sites overlapping ESC-associated H3K4me2 or H3K4me3 signals from ChIP-seq. The gray triangle indicates the position of the 20 MbMt-hypermethylated sites from ~40 to 400 bp downstream of the TSS of *HOXB-AS3* variant 3 as in Figure 4a.
